# Comparison of spleen transcriptomes of two wild rodent species reveals differences in the immune response against *Borrelia afzelii*

**DOI:** 10.1002/ece3.6377

**Published:** 2020-05-25

**Authors:** Xiuqin Zhong, Max Lundberg, Lars Råberg

**Affiliations:** ^1^ Department of Biology Lund University Lund Sweden

**Keywords:** *Borrelia*, de novo transcriptome assembly, eco‐immunology, GSEA, RNA‐Seq

## Abstract

Different host species often differ considerably in susceptibility to a given pathogen, but the causes of such differences are rarely known. The natural hosts of the tick‐transmitted bacterium *Borrelia afzelii*, which is one of causative agents of Lyme borreliosis in humans, include a variety of small mammals like voles and mice. Previous studies have shown that *B. afzelii‐*infected bank voles (*Myodes glareolus*) have about ten times higher bacterial load than infected yellow‐necked mice (*Apodemus flavicollis*), indicating that these two species differ in resistance. In this study, we compared the immune response to *B. afzelii* infection in these host species by using RNA sequencing to quantify gene expression in spleen. Gene set enrichment analysis (GSEA) showed that several immune pathways were down‐regulated in infected animals in both bank voles and yellow‐necked mice. Moreover, IFNα response was up‐regulated in *B. afzelii*‐infected yellow‐necked mice, while IL6 signaling and the complement pathway were down‐regulated in infected bank voles; differences in regulation of these three pathways between bank voles and yellow‐necked mice could thus contribute to the difference in resistance to *B. afzelii* between the species. This study provides knowledge of gene expression induced by a zoonotic pathogen in its natural host, and possible species‐specific regulation of immune responses associated with resistance.

## Graphical abstract

Previous studies have shown that *Borrelia afzelii*‐infected bank voles have about ten times higher bacterial load than infected yellow‐necked mice, indicating that these two species differ in resistance. In this study, gene set enrichment analysis (GSEA) showed that IFNα response was up‐regulated in *B. afzelii*‐infected yellow‐necked mice, while IL6 signaling and the complement pathway were down‐regulated in infected bank voles; differences in regulation of these three pathways between bank voles and yellow‐necked mice could thus contribute to the difference in resistance to *B. afzelii* between the species.

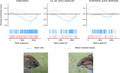

## 1 INTRODUCTION

Most pathogens can infect a number of different host species (Woolhouse, Taylor, & Haydon, 2001), but the severity of disease when infected by a given pathogen often differs considerably between host species. For example, in the case of zoonotic pathogens, natural hosts typically present little or no disease symptoms, while humans and other spillover hosts often present severe symptoms and potentially high fatality (Bean et al., 2013; Mandl et al., 2014). Such differences in susceptibility do not only occur between natural and non‐natural or spill‐over hosts, though, but also among natural hosts. For example, mortality rates differ between amphibian species when exposed experimentally to *Batrachochytrium dendrobatidis* (Gahl, Longcore, & Houlahan, 2012), and the parasitaemia of avian malaria *Haemoproteus majoris* varies among host species (Huang, Ellis, Jönsson, & Bensch, 2018). Interspecific variation in susceptibility could be a result of variation in resistance and/or tolerance (where resistance refers to variation in the ability to control pathogen replication, while tolerance means variation in ability to limit the damage of a given pathogen load; Råberg, Sim, & Read, 2007). Variation in resistance and/or tolerance can, in turn, be a result of numerous different factors, including differences in anatomy, behavior, and metabolism between host species, but divergence in immune defense likely plays the most important role (Mandl et al., 2014). Yet, there is relatively limited information on differences in immune responses to a given pathogen between host species, in particular closely related ones (but see Poorten & Rosenblum, 2016 and Palesch et al., 2018 for recent exceptions).

The tick‐transmitted bacterium *Borrelia burgdorferi* sensu lato (s.l.) is divided into 22 genospecies, of which at least three (*B. burgdorferi* sensu stricto (s.s.), *B. afzelii*, and *B. garinii*) cause Lyme borreliosis in humans (Kurtenbach et al., 2006; Waindok, Schicht, Fingerle, & Strube, 2017). *Borrelia afzelii* primarily infect rodents and *B. garinii* infect birds, while *B. burgdorferi* s.s has a wider natural host range, including both rodents and birds (Gern & Humair, 1998; Humair & Gern, 2000). In humans, untreated *Borrelia* infection may develop into diverse clinical manifestations, including neuroborreliosis, Lyme arthritis and carditis (Stanek, Wormser, Gray, & Strle, 2012), and at least some strains of laboratory mice display similar symptoms (Lin et al., 2014; Wooten & Weis, 2001). In contrast, no or limited pathology occurs in the natural hosts, like white‐footed mouse (*Peromyscus leucopus*), bank vole (*Myodes glareolus*), and yellow‐necked mouse (*Apodemus flavicollis*) (Moody, Terwilliger, Hansen, & Barthold, 1994; Zhong, Nouri, & Råberg, 2019). Previous studies have shown that the bacterial load of *B. afzelii* in infected individuals differs considerably between host species; for example, bank voles have ten times higher loads than yellow‐necked mice (Råberg, 2012; Zhong et al., 2019). Since there are no differences in prevalence of different *B. afzelii* strains between these host species (Råberg et al., 2017), the difference in resistance is most likely caused by interspecific variation in the immune response. This hypothesis is supported by the higher level of *Borrelia*‐specific antibodies found in yellow‐necked mice than in bank voles (Kurtenbach et al., 1994).

Studies of the immune response to *Borrelia* have primarily been performed in laboratory mice and humans. Briefly, signaling by pattern recognition receptors, including but not limited to toll‐like receptor 1 and 2 (TLR1 and TLR2; Oosting, Buffen, van der Meer, Netea, & Joosten, 2014), leads to production and secretion of pro‐inflammatory cytokines, like interleukin (IL) 1β, IL2, IL6, tumor necrosis factor α (TNFα), and type I interferons (IFNs) (Jones et al., 2008; Müllegger et al., 2000; Petzke et al., 2016). In addition, the complement system help clear bacteria by promoting phagocytosis through opsonization (Kurtenbach et al., 2002). A strong and effective antibody response against *B. burgdorferi* infection in mice also occurs (Connolly & Benach, 2005; McKisic & Barthold, 2000). Despite the immune response, bacteria typically disseminate from the tick bite site and colonize internal tissues (Zhong et al., 2019). Studies of both *B. burgdorferi* and *B. afzelii* have shown that infections may be chronic (persist for life), although the length of the infection may depend on bacterial strain (Humair, Rais, & Gern, 1999; Kurtenbach et al., 2006).

To investigate the immune response to *B. afzelii* in natural hosts, in particular how it differs among host species, we here compare the immune response during infections in the bank vole and yellow‐necked mouse, in the wild. To this end, we generated spleen transcriptomes of bank voles and yellow‐necked mice and compared gene expression between *B. afzelii*‐infected and *B. afzelii*‐uninfected animals.

## 2 METHODS AND MATERIALS

### 2.1 Study species and field work

The bank vole and the yellow‐necked mouse are small rodents (adult body mass 18–28 g and 24–48 g, respectively) native to Europe and Western Asia (Wilson, Mittermeier, & Lacher, 2017). In Southern Sweden, the two species occur in similar habitats, primarily deciduous woodland, and parasites and pathogens are to a large extent shared between species. For example, at our study site, both species are infested with ticks (*Ixodes ricinus*) and fleas and infected with the vector‐borne bacteria *B. afzelii, Candidatus* Neoehrlichia mikurensis, and *Bartonella* spp (Andersson & Råberg, 2011; Hellgren, Andersson, & Råberg, 2011), as well as various helminths (Clough & Råberg, 2014; X. Zhong and L. Råberg, unpublished data).

Animals for the present study were trapped at one locality (Stensoffa) in the Revinge area, 20 km east of Lund, southern Sweden, during five days in August and September 2016 using live traps (Ugglan special, Grahn AB). The habitat at the trapping locality is mixed deciduous forest dominated by beech (*Fagus sylvatica*). To minimize variation in gene expression related to for example sex, development or reproductive status, we focused on adult males (adult age indicated by the presence of a scrotum). Based on previous mark‐recapture studies of the same population (Tschirren et al., 2013; L. Råberg unpublished data), the vast majority of adult bank voles present in August‐September was born in early summer the same year (rather than being over‐wintering individuals) and were thus about three months old at the time of sampling for the present study. To avoid bias in gene expression due to short‐term variation in environmental conditions, we also made sure to collect equal numbers of the two species on each trapping day.

The spleen is an important immune organ, containing large numbers of immune cells like phagocytes and lymphocytes. The spleen is often enlarged during infection due to proliferation of lymphocytes. Gene expression in the spleen should therefore reflect the overall activity of the immune system in an individual.

Animals were transported to Stensoffa field station (300 m from the trapping site), euthanized by cervical dislocation, and spleens for RNA extraction were immediately collected and stored in RNAlater RNA Stabilization Reagent (Qiagen GmbH). Ear biopsies (one from the inner part of each ear) for determining infection status were collected and stored in 70% ethanol. Animals were weighed, and sex was determined phenotypically.

### 2.2 DNA extraction and infection status identification

Total DNA was extracted from ear biopsies following the protocol of Laird et al. (1991). To improve the accuracy of infection status, biopsies from left and right ear from each individual were prepared for DNA extraction separately. The quantity and quality of DNA were assessed by measuring the concentration of DNA and the ratio of UV absorption at 260–280 nm by Nanodrop (Thermo Fisher Scientific). Infection status was determined by quantitative PCR (qPCR) based on the *flaB* gene of *B. afzelii* as described in Råberg (2012). Animals were considered infected if at least one *flaB* gene copy per nanogram of host DNA was detected in the sample from each ear. Besides *B. afzelii,* we assessed infection status with respect to helminths (by dissecting the intestines and counting all helminths) and tick loads (*Ixodes ricinus*; by counting the number of ticks on the ears).

### 2.3 RNA extraction and sequencing

Spleens were homogenized with stainless steel beads in TissueLyser II (Qiagen). Total RNA was extracted by RNeasy Mini Kit (Qiagen, GmbH) and treated by RNase‐Free DNase Set (Qiagen, GmbH). To estimate RNA quality, the ratio of the UV absorption at 260 to 280 nm was calculated. RNA integrity and concentration (ng/μl) was measured with an Agilent Bioanalyzer (Agilent). All samples had acceptable A260/A280 ratios (2.0–2.1) and RIN (RNA Integrity Number) values (> 8.8). Samples were sent to SciLifeLab for RNA sequencing on a HiSeq2500 (Illumina) with a 2 × 126 bp setup using 'HiSeq SBS Kit v4' chemistry in 6 lanes and a HiSeq Control Software 2.2.58/RTA 1.18.64.

### 2.4 De novo transcriptome assembly and annotation

The quality of raw reads was checked with FastQC version 0.11.5 (https://www.bioinformatics.babraham.ac.uk/projects/fastqc/). Low‐quality bases in reads and adaptor sequences were removed by using Trimmomatic (Bolger, Lohse, & Usadel, 2014) with settings “2:30:10 SLIDINGWINDOW:4:5 LEADING:5 TRAILING:5 MINLEN:25”. All trimmed reads passed the quality test in FastQC. De novo transcriptome assemblies for bank vole and yellow‐necked mouse were constructed using Trinity version 2.3.2 (Grabherr et al., 2011) with default settings. To assess the completeness of the transcriptome assemblies, the number of reads that could be mapped back to the contigs was quantified. In addition, the representation of full‐length reconstructed protein‐coding genes was estimated by BLASTx version 2.6.0 (Camacho et al., 2009) of the assembled contigs against a set of manually reviewed rodent proteins (*N* = 26,560) downloaded from Swiss‐Uniprot (www.uniprot.org, 2017‐05‐13).

To reduce the redundancy in the Trinity assembly, contigs were clustered with CD‐HIT version 4.6.8 (Fu, Niu, Zhu, Wu, & Li, 2012) using a minimum sequence identity of 95% (‐c 0.95). To further reduce the redundancy and to enrich for protein‐coding sequence contigs, TransDecoder version 5.0.1 (Haas et al., 2013) was used to identify open reading frames (ORF). For each CD‐hit‐clustered group, the contig with the longest ORF was saved. To reduce potential assembly errors, reads were mapped back to the filtered contigs using RSEM version 1.3.0 (Li & Dewey, 2011), and contigs with TPM (transcripts per million mapped reads) values <1 were removed.

The assembled contigs were annotated by using BLASTx version 2.6.0 to search against house mouse (*Mus musculus*) proteins (91,244 transcripts from 22,237 unique protein‐coding genes annotated in Ensembl 87, www.ensembl.org), with an e‐value cutoff at 1e‐10. When several contigs had a best hit to the same mouse protein, the one with highest bit score was selected. Finally, the completeness of our annotated assemblies was determined by searching for 4,104 single‐copy mammalian orthologues using BUSCO v3 (Simão, Waterhouse, Ioannidis, Kriventseva, & Zdobnov, 2015).

### 2.5 Mapping approaches

To obtain estimates of gene expression that were comparable between species, we wanted to use similar approaches for both species. An annotated reference genome is available for the bank vole (Lundberg, Zhong, Konrad, Olsen, & Råberg, 2020) but not for the yellow‐necked mouse, so mapping reads from each species to its reference genome was not possible. Instead, we considered two other mapping approaches: (a) mapping reads from both voles and mice to the house mouse genome, and (b) mapping reads to de novo transcriptomes for each species.

To test the first approach, trimmed bank vole and yellow‐necked mouse reads were mapped to the house mouse genome (GRCm38) with STAR version 2.5.3 (Dobin et al., 2013). Four maximum mismatch numbers (10, 12, 25, and 50) were tested for each species. Regardless of mismatch number, mapping rates were considerably higher in yellow‐necked mouse than in bank vole (not shown). To avoid biased results because of differences in evolutionary distance between our two species and the reference genome, we therefore decided to map reads from each species to its de novo transcriptome instead.

We evaluated the second approach by comparing gene expression in bank voles when mapping to the reference genome and the de novo transcriptome. Reference genome mapping was performed with STAR version 2.5.3 followed by read count quantification using Cufflinks version 2.2.1 (Trapnell et al., 2010). Mapping to de novo transcriptomes and quantification was performed with RSEM version 1.3.0. To compare the results from mapping to the reference genome and the de novo transcriptome, we calculated the correlation of gene expression values (FPKM, Fragments Per Kilobase Million) for each individual vole.

### 2.6 Differential expression analysis within species

Differential expression between *B. afzelii‐*infected and *B. afzelii*‐uninfected animals was calculated using edgeR (Robinson, McCarthy, & Smyth, 2009) for bank voles and yellow‐necked mice separately. Counts generated from RSEM for each gene were used as input for edgeR, which normalizes counts data using trimmed mean of M‐values (TMM). Genes that are lowly expressed (<10 counts detected in more than half of the individuals) were discarded to reduce the number of tests carried out in the differential expression analysis. Genes were considered as significantly differentially expressed between groups when the |log_2_ fold change| >1 and the false discovery rate (FDR) value <0.05.

### 2.7 Gene set enrichment analysis

As a complement to the differential expression analyses, we performed gene set enrichment analyses (GSEA; Subramanian et al., 2005). This method is useful when the data are noisy, as in our case, where we compared individuals sampled in the wild, rather than from a controlled experiment. All genes were ranked based on the association between their expression and the class distinction (infected or uninfected), as measured by the sign of the fold change multiplied by the inverse of the p value (both obtained from differential expression analysis in edgeR). GSEA then uses a priori defined gene sets (such as the genes in a particular pathway), and tests whether the members of a gene set are randomly distributed throughout the ranked list of all expressed genes, or enriched at the top or bottom. If genes in a gene set are enriched at either the top (up‐regulated) or bottom (down‐regulated), the gene set is considered to be related to the phenotypic difference between samples. In the software, the GSEAPreranked module was used to calculate a running enrichment score for each gene in our ranked gene list.

We used the Hallmark gene sets from The Molecular Signatures Database v7.0 (Liberzon et al., 2015), which summarize and represent specific well‐defined biological states or processes, for running GSEA. Nominal *p* value < .05 and adjusted *p* value < .25 were considered as statistically significant (Subramanian et al., 2005). We were particularly interested in gene sets, such as IL2_STAT5 signaling, IL6_JAK_STAT3 signaling, TNFα_via_NFκB signaling, interferon alpha (IFNα) response, and inflammatory response, which could be expected to be highly expressed in the spleen and cover immunological pathways known to be involved in the immune response to *Borrelia* infection based on studies of laboratory mice and humans (Jones et al., 2008; Kurtenbach et al., 2002; Müllegger et al., 2000; Petzke et al., 2016). IL2 is a cytokine involved in T‐cell proliferation and differentiation (Murphy & Weaver, 2016). IL6 and TNFα are pro‐inflammatory cytokines, and have a wide spectrum of effects involved in the response to infection by pathogens, including extracellular bacteria like *Borrelia*. IFNα is primarily known as an anti‐viral cytokine, but recent studies found it is also involved in responses against bacteria (Kovarik, Castiglia, Ivin, & Ebner, 2016).

## 3 RESULTS

### 3.1 RNA sequencing and quality control

Thirty‐five samples (18 bank voles and 17 yellow‐necked mice) were sequenced, resulting in total ~1.44 billion reads with between 29.42 and 54.91 million reads per individual. After removing low‐quality bases and adaptors, the data consisted of ~1.35 billion trimmed and paired reads.

### 3.2 De novo assembled transcriptomes

The assembly of the bank vole transcriptome resulted in 842,299 contigs (presumptive transcripts) grouped in 641,425 clusters (presumptive genes), with a mean length of 899 bp and N50 of 1,799 bp. The yellow‐necked mouse de novo assembled transcriptome consisted of 761,841 contigs and 565,794 clusters with a mean length of 976 bp and N50 of 2,022 bp. When mapping back the reads used in the assembly, both of the assemblies showed a high proportion (>95%) of properly aligned read pairs (Table 1). The bank vole and yellow‐necked mouse assemblies also showed similar numbers of matched rodent proteins (19,065 and 18,819) (Table 1).

**Table 1:** Statistics of de novo transcriptome assemblies and annotations in bank vole and yellow‐necked mouse

	Bank vole	Yellow‐necked mouse
Number of contigs	842,299	761,841
Number of clusters	641,425	565,794
Contig N50 (bp)	1,799	2,022
Average length of contigs (bp)	899	976
Properly aligned pairs	656,999,914 (95.81%)	641,223,018 (96.57%)
Matched proteins (Rodentia)	19,065	18,819
Proteins with full‐length CDs	10,544 (55.3%)	10,978 (58.33%)
Contigs after filtering by		
CD‐HIT	660,968	588,508
TransDecoder	117,706	114,379
>1 TPM	31,944	32,238
Annotated contigs	19,147	19,181
Nonredundant annotated contigs	13,631	13,744
Complete and single‐copy BUSCOs	2,506 (61.1%)	2,589 (63.1%)
Complete and duplicated BUSCOs	14 (0.3%)	22 (0.5%)
Fragmented BUSCOs	924 (22.5%)	840 (20.5%)
Missing BUSCOs	660 (16.1%)	653 (15.9%)

### 3.3 Removal of redundant contigs

Using a similarity‐based clustering with CD‐HIT and selecting the longest contig in each cluster, the number of contigs were reduced to 660,968 and 588,508 in the bank vole and yellow‐necked mouse, respectively. Selecting the longest ORF for each of the CD‐HIT clusters resulted in 117,706 contigs for bank vole and 114,379 contigs for yellow‐necked mouse. Following the removal of contigs with low expression levels (TPM < 1), the transcriptome assemblies consisted of 31,944 contigs for bank vole and 32,238 contigs for yellow‐necked mouse (Table 1).

### 3.4 Gene annotation

In the filtered transcriptome assemblies, 19,147 contigs in the bank vole and 19,181 contigs in the yellow‐necked mouse could be matched to house mouse proteins. Selecting the contig with the highest bit score to a specific mouse gene resulted in the retention of 13,631 contigs and 13,744 contigs in bank vole and yellow‐necked mouse, respectively (Table 1). The two nonredundant transcriptome assemblies showed similar levels of complete and partial mammal single‐copy orthologues (Table 1).

### 3.5 Mapping to de novo assembly versus reference genome

When mapping bank vole reads to the reference genome, 78.05% of reads were mapped and assigned to a unique gene, while the alignment rate to the de novo transcriptome was 51.44%. Gene expression values (log_2_ FPKM) estimated from the two approaches were highly correlated within each individual (r: 0.91 – 0.93). Therefore, we decided to use de novo transcriptome assemblies as reference for mapping reads in both species.

### 3.6 Infection status

**Figure 1:**
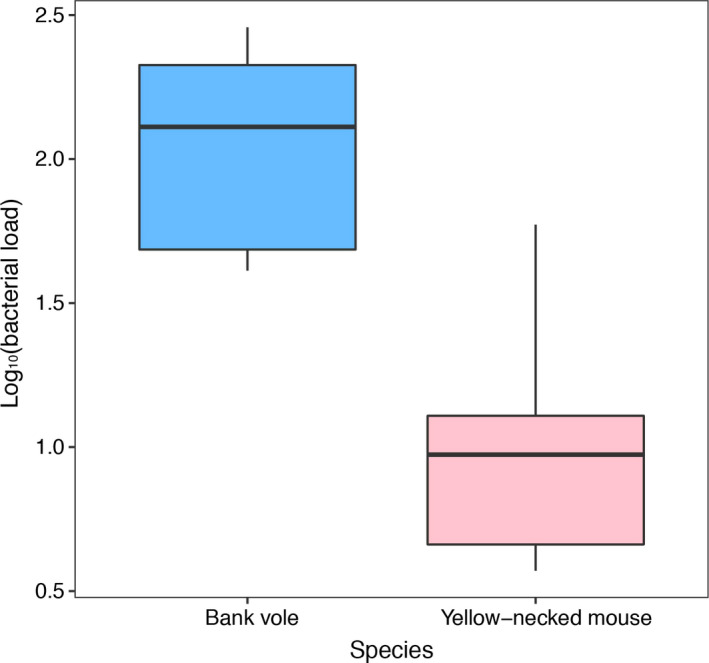
Box plot of bacterial loads of *Borrelia afzelii* in infected bank voles and yellow‐necked mice. The box plots indicate the median, first, and third quartiles, and range of the data. Bacterial loads were quantified as the number of spirochetes (as measured by qPCR targeting the flaB gene) per nanogram of host DNA (as measured by Nanodrop)

Of the eighteen bank voles, seven were infected and nine were uninfected, while two had ambiguous infection status (very low infection intensity in one ear). Of the seventeen yellow‐necked mice, eight were infected and six were uninfected, while three had ambiguous infection status. Samples with ambiguous infection status were excluded from further analyses. The bacterial load of *B. afzelii* in bank voles was 10‐fold higher than in yellow‐necked mice (*F*_1,13_ = 20.5, *p* = .004, Figure 1).

All individuals, except one bank vole were infested with tick larvae (bank voles: 1–49 ticks/individual; yellow‐necked mice: 3–42 ticks/individual). Helminth infections (mainly *Heligmosomoides* sp., but also a few other species) were detected in 15 out of 18 bank voles (range 1–18 worms/individual), and 13 out of 17 yellow‐necked mice (range 1–19).

### 3.7 Differentially expressed genes within species

To compare gene expression in infected and uninfected voles and mice, we first performed differential expression analyses in each species separately. After filtering out genes with low expression (<10 counts detected in more than half of the individuals), 12,185 genes of bank vole and 12,282 genes of yellow‐necked mouse remained.

**Figure 2:**
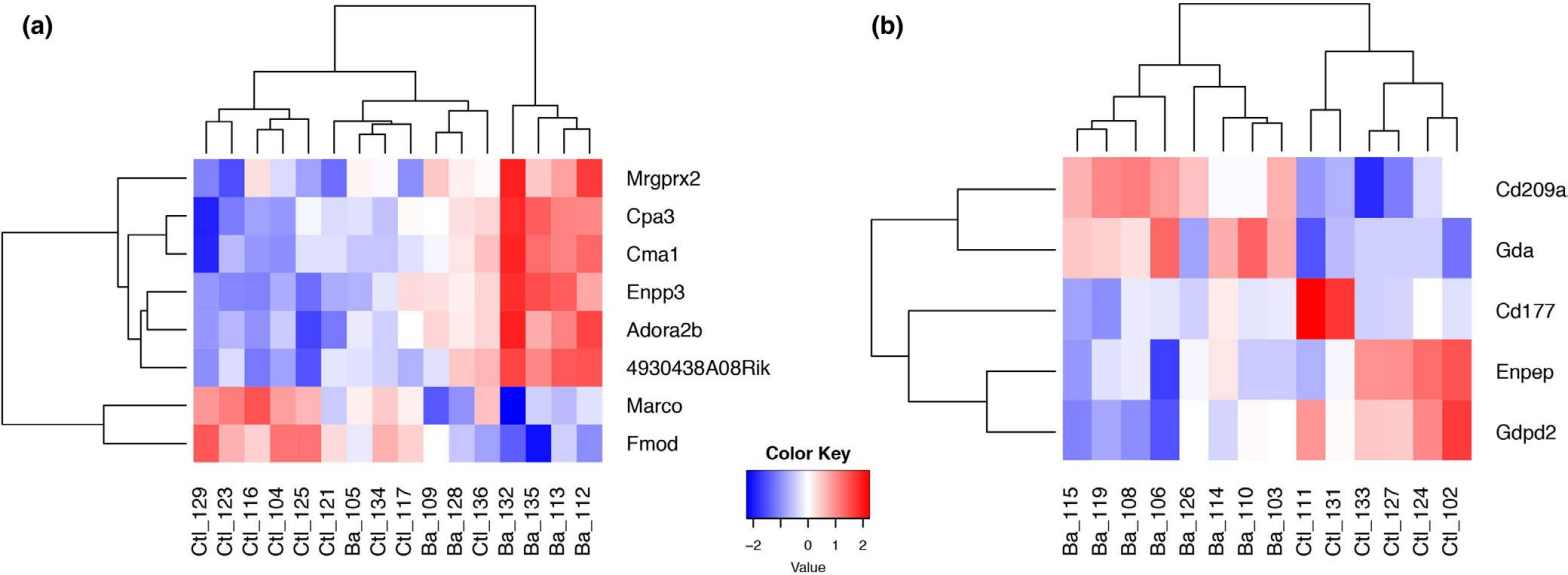
Heat maps and hierarchical clustering of significantly differentially expressed genes between *Borrelia afzelii*‐infected and *B. afzelii*‐uninfected individuals. (a) Bank vole; (b) Yellow‐necked mouse. Ba_XXX are infected individuals, and Ctl_XXX are uninfected

In bank voles, eight genes were differentially expressed between infected and uninfected individuals. Six of these were up‐regulated in *B. afzelii‐*infected bank voles, while two genes were down‐regulated (Figure 2a, Table 2). However, clustering of samples based on the similarity of their gene expression pattern did not group infected and uninfected animals in separate clusters (Figure 2a).

**Table 2:** Differentially expressed genes in spleens infected with *B. azfelii* in bank voles and yellow‐necked mice

Name	Description	logFC	*p* Value	FDR
*B. afzelii*‐infected versus uninfected bank voles				
4930438A08Rik	RIKEN cDNA 4930438A08 gene	2.27	7.02E−06	0.031
Adora2b	Adenosine A2b receptor	1.18	1.65E−05	0.031
Mrgprx2	MAS‐related GPR, member X2	1.62	1.84E−05	0.031
Cma1	Chymase 1, mast cell	2.08	2.05E−05	0.031
Cpa3	Carboxypeptidase A3, mast cell	1.80	2.21E−05	0.031
Enpp3	Ectonucleotide pyrophosphatase/phosphodiesterase 3	1.89	2.31E−05	0.031
Marco	Macrophage receptor with collagenous structure	−3.16	1.20E−05	0.031
Fmod	Fibromodulin	−1.45	1.52E−05	0.031
*B. afzelii*‐infected versus uninfected yellow‐necked mice				
Cd209a	CD209a antigen	1.97	1.15E−06	0.014
Enpep	Glutamyl aminopeptidase	−3.01	6.57E−06	0.017
Gda	Guanine deaminase	1.32	3.06E−06	0.017
Gdpd2	Glycerophosphodiester phosphodiesterase domain containing 2	−1.03	6.83E−06	0.017
Cd177	CD177 antigen	−4.07	1.00E−05	0.018

Note

LogFC, log_2_‐transformed fold change, positive value means higher expression in the *Borrelia afzelii*‐infected individuals, negative value means lower expression in the *B. afzelii*‐infected individuals

In yellow‐necked mice, five genes with significantly differential expression were found; three genes had higher expression in *B. afzelii‐*infected animals than in controls, while two genes had lower expression (Figure 2b, Table 2). The infected and uninfected yellow‐necked mice clustered separately according to gene expression patterns.

### 3.8 Gene set enrichment analysis

**Figure 3:**
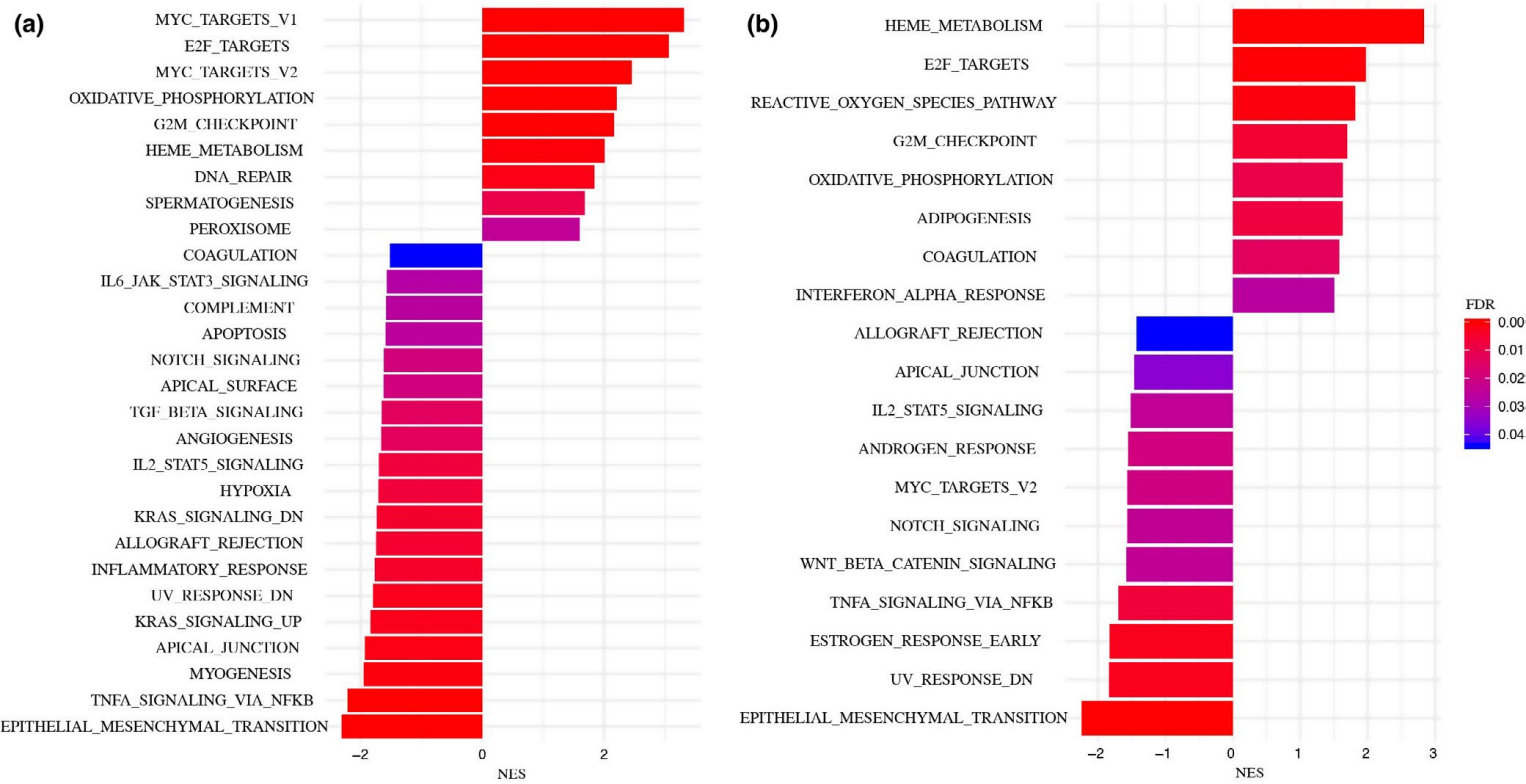
Gene set enrichment analysis of hallmark gene sets performed on individuals infected with *Borrelia afzelii* compared with the uninfected individuals in bank voles (a) and yellow‐necked mouse (b). Normalized enrichment scores (NES) for each gene set are shown with bars, gradient of the color represents false discovery rate (FDR). Positive and negative NES represent up‐regulated gene set and down‐regulated gene sets, respectively

**Figure 4:**
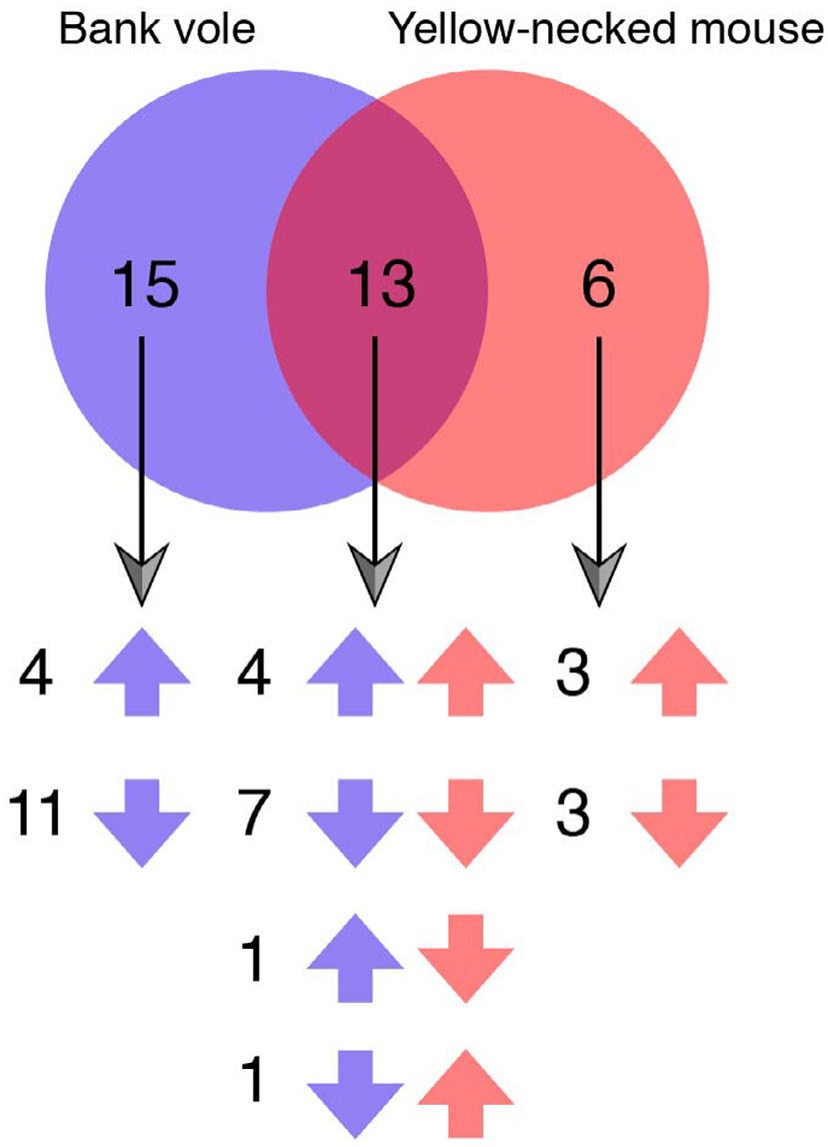
Venn diagram of hallmark gene sets that were differently regulated in *Borrelia afzelii‐*infected versus uninfected bank voles and yellow‐necked mice. Up arrows represent gene sets up‐regulated in *B. afzelii*‐infected individuals; down arrows represent gene sets down‐regulated in *B. afzelii*‐infected individuals

To further explore the effects of *B. afzelii* infection on gene expression, we performed GSEA with all 50 Hallmark gene sets. In bank voles, nine gene sets were up‐regulated in *B. afzelii‐*infected individuals compared with uninfected, while nineteen gene sets were down‐regulated (Figure 3a). In *B. afzelii‐*infected yellow‐necked mice, eight gene sets were up‐regulated and eleven were down‐regulated (Figure 3b). Among the thirteen gene sets that were up‐ or down‐regulated in both species, eleven had the same pattern of regulation in both species, while two gene sets were regulated in opposite ways (Figure 4). It should be noted that some of the gene sets that were up‐ or down‐regulated in infected versus uninfected individuals have no obvious function in the spleen (e.g., spermatogenesis, UV‐response); those cases presumably reflect differential expression of highly pleiotropic genes that are also involved in pathways expressed in the spleen.

To better understand the immune response against *B. afzelii,* we focused on the seven immunological Hallmark gene sets. Of these, six were differentially regulated between infected and uninfected individuals in at least one of the species (all but IFNγ response). Each of these six gene set consists of 87–200 genes involved in a specific immune pathway. In bank vole, 73%–86% of the genes in each gene set were represented in the de novo assembly, while 72%–86% were represented in the yellow‐necked mouse assembly. In each gene set, 70%–81% of the genes were shared between bank vole and yellow‐necked mouse (Table 3).

**Table 3:** Number of genes for each gene set, shared genes between species and unique core genes in each species

Gene sets	No. of genes in gene set	No. of matched genes in BV	No. of matched genes in YNM	No. of genes matched in BV &YNM	No. of core genes in BV not matched in YNM	No. of core genes in YNM not matched in BV
TNFA_SIGNALING_VIA_NFKB	200	167	167	157	9	1
IL2_STAT5_SIGNALING	200	172	171	162	5	1
INFLAMMATORY_RESPONSE	200	160	154	146	4	1
IL6_JAK_STAT3_SIGNALING	87	69	68	63	4	0
INTERFERON_ALPHA_RESPONSE	97	77	75	69	2	5
COMPLEMENT	200	146	143	138	2	2

Abbreviations: BV, bank vole; YNM, yellow‐necked mouse.

**Figure 5:**
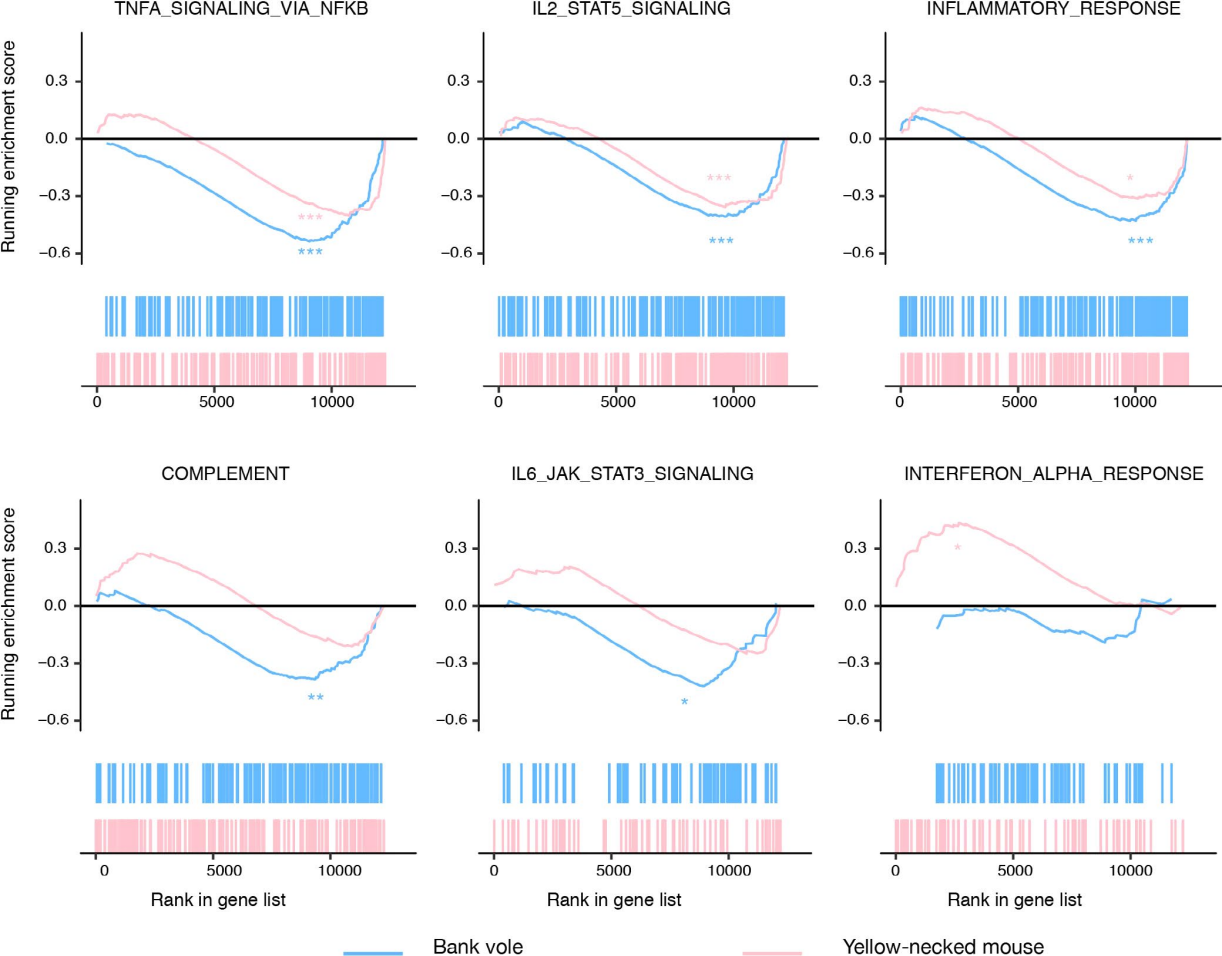
Gene set enrichment plots of immunological Hallmark gene sets differently regulated in response to *Borrelia afzelii* infection in at least one of the two host species. For each plot, the top portion shows the running enrichment score (ES) for the gene set as the analysis walks down the ranked list, the bottom portion shows where the members of the gene set appear in the ranked list of genes. In each enrichment plot, the score at the peak of the plot (the score furthest from 0.0) is the enrichment score for the gene set. *p < .05, **p < .01, ***p < .001

Three gene sets (IL2_STAT5 signaling, TNFα_via_NFκB signaling and inflammatory response), showed significant negative enrichment scores in both species, indicating that these three gene sets were down‐regulated in *B. afzelii‐*infected individuals in both host species (Figure 5, Table 4). Two gene sets (complement system and IL6_JAK_STAT3 signaling) were down‐regulated in *B. afzelii‐*infected bank voles, while there was no difference in yellow‐necked mice. Finally, the gene set IFNα response was up‐regulated in *B. afzelii‐*infected yellow‐necked mice, but not in bank voles (Figure 5, Table 4).

**Table 4:** Enrichment of six immunological gene sets between phenotypes (*Borrelia afzelii‐*infected and uninfected)

Gene sets	Matched size	ES	NOM p‐val	FDR q‐val	Rank at max
*B. afzelii*‐infected versus uninfected bank voles					
TNFA_SIGNALING_VIA_NFKB	167	−0.54	0	0	3,121
INFLAMMATORY_RESPONSE	160	−0.43	0	0.001	2,161
IL2_STAT5_SIGNALING	172	−0.41	0	0.002	2,546
COMPLEMENT	146	−0.39	0.001	0.008	2,833
IL6_JAK_STAT3_SIGNALING	69	−0.43	0.01	0.009	3,235
INTERFERON_ALPHA_RESPONSE	77	−0.21	0.868	0.891	3,278
*B. afzelii*‐infected versus uninfected yellow‐necked mice					
INTERFERON_ALPHA_RESPONSE	75	0.44	0.012	0.027	2,678
COMPLEMENT	143	0.28	0.343	0.45	1769
TNFA_SIGNALING_VIA_NFKB	167	−0.41	0	0.007	1518
IL2_STAT5_SIGNALING	171	−0.36	0	0.025	2,642
INFLAMMATORY_RESPONSE	154	−0.32	0.034	0.093	2,205
IL6_JAK_STAT3_SIGNALING	68	−0.27	0.443	0.509	1,061

Note

Abbreviation: ES, enrichment score.

Positive ES indicates gene set is up‐regulated in *B. afzelii‐*infected individuals; negative ES indicates gene set is down‐regulated in infected individuals.

**Figure 6:**
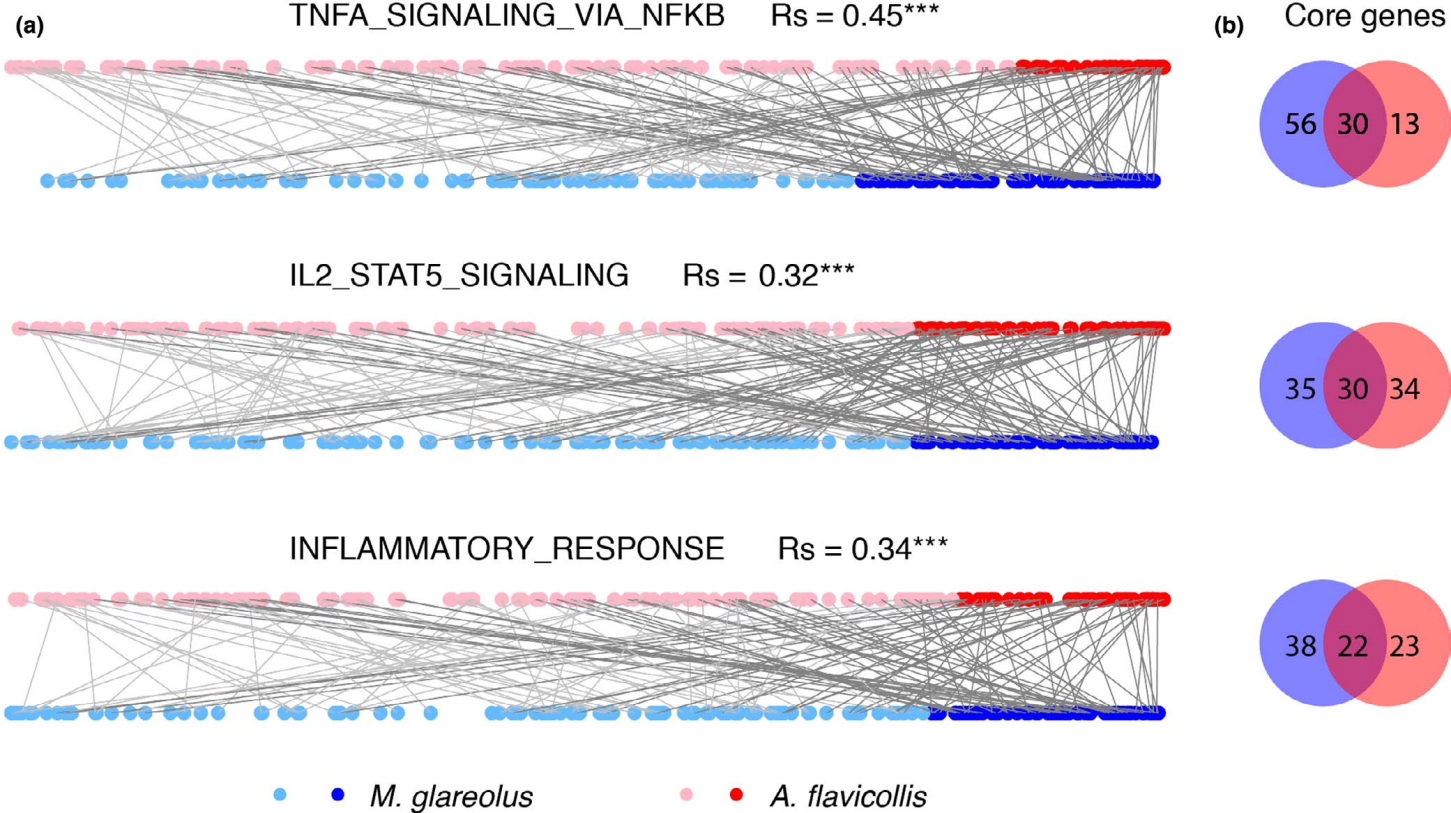
Similarity of regulation of individual genes in gene sets that were down‐regulated in *Borrelia afzelii*‐infected individuals in both bank voles and yellow‐necked mice. (a) Correlation of gene ranks between bank voles and yellow‐necked mice; (b) Venn diagrams showing the number of overlapping core genes from each gene set between two species. Rs, Spearman's correlation coefficient. *p < .05, **p < .01, ***p < .001

Three of the immunological Hallmark gene sets were down‐regulated in both species (IL2_STAT5 signaling, TNFα_via_NFκB signaling and inflammatory response). To investigate to what extent genes within each of these gene sets were regulated in the same way in both species, we performed two analyses. First, we calculated the correlation between the rankings of genes in the two species. For all three gene sets, rankings were moderately positively correlated (Figure 6a). Secondly, we calculated to what extent “core genes” were shared between species. Core genes are the genes at the leading edge of a gene set, that is the subset of genes that contribute most to the enrichment score of gene set. As shown in Figure 6b, for each gene set, the core genes were shared to 27%–30%.

## 4 DISCUSSION

In this study, we analyzed and compared the immune responses to *B. afzelii* in two natural host species, through RNA sequencing of spleen transcriptomes of naturally infected and uninfected individuals. GSEA showed that immunological gene sets were generally down‐regulated in infected animals. Moreover, GSEA indicated that three immunological gene sets (response to IFNα, IL6 signaling and complement system) were up‐ or down‐regulated in response to *B. afzelii* infection in only one of the species.

One important limitation of our study is that we do not know when animals were infected; some might have the acquired the infection recently while other carried it for months. Since both host species were sampled at the same site (and thereby have been exposed to ticks for the same amount of time), it is unlikely differences in infection length between species biased the results; rather, variation in infection length among individuals is likely to have introduced noise. Another limitation is that we have little information about which other pathogens the animals in the present study were infected with. Our previous studies of the same populations have shown that the bank vole and yellow‐necked mouse share a number of important rodent pathogens, although the prevalence differs somewhat between host species (Andersson & Råberg, 2011; Clough & Råberg, 2014; Hellgren et al., 2011). In addition, there could of course be other pathogens that primarily occur in one of the host species. Such differences in prevalence of other pathogens could potentially bias our analyses of the response to *B. afzelii*.

Studying transcriptomes of nonmodel species without a reference genome has become feasible with the advent of RNA‐sequencing, where de novo transcriptome assembly can be used when a reference genome is not available (Carruthers et al., 2018; Castellanos‐Martínez, Arteta, Catarino, & Gestal, 2014; Lamanna, Kirschbaum, Waurick, Dieterich, & Tiedemann, 2015). To estimate the accuracy of gene expression level quantified by using de novo assembly as reference, we compared gene expression when mapping to the bank vole reference genome and the de novo transcriptome assembly. The correlations between the two approaches were consistently strong. High consistency between gene expression estimates using reference genome and de novo assembly was also reported in previous studies (e.g., Nookaew et al., 2012).

Our analyses revealed only a handful differentially expressed genes between *B. afzelii*‐infected and *B. afzelii*‐uninfected animals. Nevertheless, several of the differentially expressed genes have clear roles in the immune system. *CD209A* (up‐regulated in infected mice) is a pattern recognition receptor expressed by innate immune cells; *CD177* (down‐regulated in infected mice) is a surface glycoprotein that plays a role in activation of neutrophils; *MRGPRX2*, *CPA3, and CMA1* (all up‐regulated in infected voles) are primarily expressed in mast cells; and *MARCO* (down‐regulated in infected voles) is a scavenger receptor expressed by innate immune cells and involved in recognition of bacteria (Bateman, 2019). However, it is obviously difficult to draw any general conclusions regarding which pathways are involved based on such a small number of differentially expressed genes. The low number of differentially expressed genes is possibly caused by that animals with *B. afzelii* infections were in different stages of the infection, and/or that some of the animals were infected with other pathogens (such as, “*Candidatus* Neoehrlichia mikurensis,” *Bartonella* spp. and helminths (Buffet et al., 2012; Andersson, Bartkova, Lindestad, & Råberg, 2013; Grzybek et al., 2015)), all of which are common in the population studied here, and may trigger partly the same immune pathways as *B. afzelii* infection. These and other factors undoubtedly contribute noise to our data set. Analyses of gene expression in tissues preferentially colonized by *B. afzelii* but not other pathogens (e.g., skin, joints) might reveal a larger number of differentially expressed genes between *B. afzelii‐*infected and *B. afzelii*‐uninfected animals.

Gene set enrichment analysis is considered more powerful in case of noisy data sets from natural populations. Indeed, the GSEA revealed that genes involved in IL2 and TNFα signaling and inflammatory response were down‐regulated in *B. afzelii‐*infected animals, in both bank voles and yellow‐necked mice. Previous studies of humans and laboratory mice have found that expression of these pro‐inflammatory cytokines in skin and blood is up‐regulated during the early phase of *Borrelia* infection (Bouquet et al., 2016; Jones et al., 2008; Müllegger et al., 2000; Zlotnikov et al., 2017). In contrast, expression of for example *TNFα* in tissues from late‐stage *Borrelia* infection in mice was reduced (Hodzic, Feng, & Barthold, 2013). Moreover, expression of inflammatory cytokines in response to in vitro stimulation with LPS of blood from late‐stage borreliosis patients was suppressed (Diterich, Härter, Hassler, Wendel, & Hartung, 2001). Thus, the down‐regulation of pro‐inflammatory cytokine expression we observed in naturally infected hosts also occurs during the chronic stage of *Borrelia* infection in non‐natural hosts.

Previous studies have found that the bacterial load is about ten times higher in *B. afzelii‐*infected bank voles than infected yellow‐necked mice, indicating that mice are more resistant (Råberg, 2012; Zhong et al., 2019). The same pattern was found in the present study, despite a relatively small data set. The GSEA showed that the main differences between voles and mice in the immune response to *B. afzelii* is that the complement system and IL6 signaling is down‐regulated in infected voles, while IFNα response is up‐regulated in infected mice. Thus, differences in regulation of these pathways between bank voles and yellow‐necked mice potentially contribute to the difference in resistance to *B. afzelii* between the species.

The complement system plays an important role in resistance against bacteria, including *Borrelia* (Kurtenbach et al., 2002). The key proteins involved in the complement system (C2, C3, C4 etc) are mainly synthesized in the liver, but some of these genes are highly expressed also in the spleen, in particular the genes encoding the different components of C1 (at least in humans; GTEx Consortium, 2015). Moreover, the Hallmark complement gene set comprises 200 genes, and thus includes a large number of genes besides the ca. 30 traditional complement proteins. Hence, it is not surprising to find differences in expression of complement genes in the spleen.

IL6 is one of the inflammatory cytokines required for the development and maintenance of the T helper 17 subset, which is important in defense against infections with extracellular bacteria, like *Borrelia* (Murphy & Weaver, 2016). IL‐6 was the predominant cytokine in skin lesions (“erythema migrans”) of Lyme borreliosis patients and is strongly induced in *Borrelia‐*stimulated human monocytes in vitro (Cervantes et al., 2011; Salazar et al., 2003).

Type I interferons (including IFNα) are traditionally regarded as an important component of the defense against viral infections, but they also play a role in the response to bacterial infections (Box x & Cheng, 2016). Indeed, expression of type I interferons and related genes was strongly induced by *B. burgdorferi* in human peripheral blood mononuclear cells (PBMCs; i.e., lymphocytes and monocytes) and laboratory mice (Cervantes et al., 2011; Hastey, Ochoa, Olsen, Barthold, & Baumgarth, 2014; Love, Schwartz, & Petzke, 2014; Petzke, Brooks, Krupna, Mordue, & Schwartz, 2009). *IFIT2, ISG20,* and *DDX60* are genes in the IFNα gene set with opposite regulation during *B. afzelii* infection in bank vole and yellow‐necked mice (highly expressed in infected mice compared with the uninfected mice; lowly expressed in infected voles). These genes are thus candidate genes for further studies of the causes of the difference in immune response against *B*. *afzelii* between bank voles and yellow‐necked mice.

The only immunological Hallmark gene set that was not differentially regulated in infected and uninfected individuals in at least one of the species was IFNγ response. The lack of effect of *B. afzelii* infection on this gene set is consistent with the fact that IFNγ is primarily involved in responses to viruses and intracellular bacteria.

Besides the canonical immune gene sets, there were some other gene sets potentially involved in the immune response that were differently regulated between infected and uninfected individuals. For example, notch signaling (down‐regulated in infection animals in both species) regulates T‐cell differentiation together with other signaling pathways, such as IL2 and type I IFN (Amsen, Helbig, & Backer, 2015). Moreover, epithelial‐mesenchymal transition (down‐regulated in infected animals in both species), which leads to formation of multipotent stromal cells, is a common consequence of inflammation and is involved in for example wound healing (Kalluri & Weinberg, 2009).

To conclude, the present study provides the de novo spleen transcriptome assemblies for two non‐model species, bank vole, and yellow‐necked mouse and reveals some general patterns as well as differences between host species in the immune response against *B. afzelii* infection in naturally infected hosts in the wild. Specifically, several immune pathways were down‐regulated during *B. afzelii* infection in both bank voles and yellow‐necked mice, something which might contribute to their role as reservoir hosts. Moreover, differences in regulation of the complement system, IL6 signaling, and IFNα response may contribute to differences in resistance to *B. afzelii* between bank voles and yellow‐necked mice. We would like to stress, though, that controlled infection experiments are required to confirm the present analyses and provide a more detailed picture of the response to *B. afzelii* in these host species.

## CONFLICT OF INTEREST

None declared.

## AUTHOR CONTRIBUTION

**Xiuqin Zhong:** Conceptualization (equal); Data curation (lead); Formal analysis (lead); Investigation (equal); Methodology (equal); Project administration (lead); Writing‐original draft (lead). **Max Lundberg:** Conceptualization (supporting); Data curation (supporting); Formal analysis (supporting); Methodology (supporting); Writing‐review & editing (supporting). **Lars Råberg:** Conceptualization (equal); Formal analysis (supporting); Funding acquisition (lead); Investigation (equal); Methodology (equal); Project administration (supporting); Supervision (lead); Writing‐review & editing (lead).

## Data Availability

The sequence data have been deposited in the Sequence Read Archive (SRA) database at the National Center for Biotechnology Information (NCBI) under the BioProject PRJNA556160. The annotated de novo assemblies and the data sets used for differential expression analyses and GSEA have been provided at Dryad (https://doi.org/10.5061/dryad.t1g1jwt02).
